# Long-Term Antipsychotic Effectiveness and Comparison of the Efficacy of Monotherapy and Polypharmacy in Schizophrenia: A 3-Years Follow-Up “Real World” Study in China

**DOI:** 10.3389/fphar.2022.860713

**Published:** 2022-06-13

**Authors:** Lei Zhang, Sidi He, Luyao He, Wenjuan Yu, Shen He, Yange Li, Yimin Yu, Qingshan Zheng, Jingjing Huang, Yifeng Shen, Huafang Li

**Affiliations:** ^1^ Department of Shanghai Mental Health Center, Shanghai Jiao Tong University School of Medicine, Shanghai, China; ^2^ Department of Psychiatry, First Affiliated Hospital of Zhengzhou University, Zhengzhou, China; ^3^ Center for Drug Clinical Research, Shanghai University of Traditional Chinese Medicine, Shanghai, China

**Keywords:** schizophrenia, time to discontinuation, treatment discontinuation rates, monotherapy, polypharmacy

## Abstract

**Background:** Discontinuation of antipsychotic treatment is a common problem in patients with schizophrenia and could reduce the effectiveness of treatment. Time to discontinuation (TTD) is one of the indicators of compliance and may also be an effective indicator of medication efficacy. The aim of the study was to compare the clinical effectiveness of quetiapine, olanzapine, risperidone, and aripiprazole in the real-world treatment of schizophrenia with 3-years follow-up.

**Method:** A multi-center, open, cohort, prospective, real-world study was conducted. 706 patients were analyzed without intervention in medication selection and use, followed up for 3 years. Kaplan-Meier survival curves were used to draw the treatment discontinuation rates (TDR) curves at each time point. Cox proportional hazard regression model was used to assess the relative risk of TTD of antipsychotics.

**Results:** There was a significant difference among monotherapy groups in all-cause antipsychotic treatment discontinuation (*p* = 0.0057). Among the four medications, the TDR of risperidone was the highest. Compared with polypharmacy, except for aripiprazole, the TDR of other three monotherapy medications were lower than that of polypharmacy, and olanzapine was statistically different (*p* = 0.0325). The cox regression analysis showed that after correction of Hochberg with multiple tests, only olanzapine had a relative risk lower than risperidone (*p* < 0.0083).

**Conclusions:** The findings indicated that risperidone monotherapy and polypharmacy had the highest TDR and the shortest TTD. Olanzapine monotherapy had a relative risk lower than risperidone and was superior to polypharmacy.

## Introduction

Schizophrenia is a chronic and serious neuropsychiatric disorder that often occurs in young adults, affecting approximately 1% of the worldwide population (Stone et al., 2010). At present, atypical antipsychotic drugs have become the first line drug treatment for people with schizophrenia (Lehman et al., 2004). Discontinuation of antipsychotic treatment is a common problem in patients with schizophrenia. Several studies have shown that discontinuation of atypical antipsychotics could reduce the effect of treatment (Mojtabai et al., 2003), increase the risk of hospitalization and financial burden (Thieda et al., 2003; Weiden et al., 2004). It was even thought that a minimum of 10 days of discontinuation would lead to relapse (Weiden et al., 2004).

Despite recognizing the importance of continuous antipsychotic treatment, only about 50% of patients with schizophrenia were adherent to antipsychotic treatment (Byerly et al., 2007; El-Missiry et al., 2015). Time to discontinuation (TTD) is one of the indicators of compliance and also be an effective indicator of efficacy, because it reflects the judgment of patients and clinicians about the efficacy, safety and tolerability of the medications (Kreyenbuhl et al., 2011). The Clinical Antipsychotic Trial of Intervention Effectiveness (CATIE) is the first large-sample multi-center prospective study with TTD as the primary efficacy indicator (Lieberman et al., 2005). It compared the efficacy of olanzapine, risperidone, quetiapine, ziprasidone and perphenazine, and concluded that olanzapine was the most effective in terms of treatment discontinuation rates (TDR) and TTD (Lieberman et al., 2005). A 3-years follow-up of 102 patients with first-episode psychosis patients found differences in the treatment discontinuation rates among aripiprazole, ziprasidone, and quetiapine (Gómez-Revuelta et al., 2018). A recent multi-center study conducted a 1-year follow-up of 569 first-episode patients with schizophrenia. There were no significant differences among the risperidone olanzapine and aripiprazole in terms of antipsychotic treatment discontinuation (Cheng et al., 2019).

For the medication treatment of schizophrenia, authoritative clinical treatment guidelines recommend the monotherapy of antipsychotic therapy. However, in clinical practice, polypharmacy of antipsychotic prevalence rates were often high. A longitudinal database study found that the prevalence rate of antipsychotic polypharmacy was 12.7% in China and 19.9% in Japan (Qiu et al., 2018). In fact, there was little evidence about the relative clinical efficacy of monotherapy for schizophrenia (Essock et al., 2009). A nationwide cohort study found that combining aripiprazole with clozapine was associated with the lowest risk of rehospitalization, being superior to clozapine, the monotherapy associated with the best outcomes, with a difference of 14% (Tiihonen et al., 2019). There was also a meta-analysis that suggests that polypharmacy may be superior to monotherapy in terms of maintenance therapy (Galling et al., 2017). Some people believed that although the polypharmacy was more effective, it also had more economic expenses. It was usually recommended as a last resort in the guidelines (Rupnow et al., 2007). While there was still a lack of studies in China on comparing the effectiveness of atypical antipsychotics between polypharmacy and monotherapy.

So we amied to study the issue with TTD and TDR as the primary indicators to evaluate the long-term clinical effects of four commonly used atypical antipsychotics (quetiapine, olanzapine, risperidone, and aripiprazole) in patients with schizophrenia. Meanwhile, the long-term clinical effects of monotherapy and polypharmacy of these four medications were also compared.

## Methods

### Study Design

The data for this study were obtained from An Observational Safety and Related Factors Study on Atypical Antipsychotics Long-term Treatment in Chinese Patients with Schizophrenia (Protocol ID: SALT-C). Its rationale, design, and methods have been described previously (Zhang et al., 2019; Yu et al., 2021). The sample size of each center is presented in Supplementary Figure S1. This was a multi-center, open, cohort, prospective, real-world study for evaluating efficacy and safety of antipsychotics.

There were 8 visits in total, including the baseline period, 12, 26, 52, 78, 104, 130, and 156 weeks. The enrolled patients continued their existing treatment plan and based on the principle of the most suitable dose maintenance treatment to adjuste according to the actual clinical situation. Benzodiazepines, zolpidem or zopiclone can be combined for insomnia. Anticholinergic drugs can be used for extrapyramidal adverse reactions, and antidepressants for depressive symptoms. All combined medications have been recorded in the case report form. The positive and negative syndrome scale (PANSS) was used to assess the severity of the symptoms of the subjects (Kay et al., 1987).The personal and social performance scale (PSP) was used to assess the social function of patients (Qiao et al., 2017).

### Subjects

This analysis included the patients with schizophrenia (DSM- IV) who received quetiapine, olanzapine, risperidone, and aripiprazole monotherapy or polypharmacy, which was commonly used in China. The exclusion criteria included substance dependency, dementia, mental retardation, and Axis I or II significant physical illness. We recorded the time and dose of medication. Only one of the four atypical antipsychotics mentioned above and the other atypical antipsychotics (quetiapine, olanzapine, risperidone, aripiprazole, ziprasidone, paliperidone, amisulpride, perospirone, and clozapine) are included in the polypharmacy group (addition of a second atypical antipsychotic to the existing one; patients who used more than two atypical antipsychotics simultaneously or combination with typical antipsychotic drugs were not investigated in the analysis).

### Outcome Measures

The principal outcome measure was all-cause discontinuation defined by the following events: discontinuation of treatment, switching to other antipsychotics, initiation of concomitant new antipsychotic as add-on therapy, discontinuation of either one of the medications in the polypharmacy of antipsychotics, lost to follow-up with missing data, or death due to any reason. For inpatients, the inpatient medical records shall prevail, and for outpatients, the comprehensive judgment shall be based on the narratives of the patients and their guardians and the outpatient medical records. If the patient’s medication adherence was found to be poor in a certain month, and the patient did not improve in the next month after being urged, the first month was determined as the discontinuation of the treatment. Any reason for the relapse of psychiatric symptoms can also be determined as discontinuation. The TTD was calculated based on the point of discontinuation. Relapse is determined by meeting any of the following criteria (Csernansky et al., 2002): (1) aggravation or repeated need for psychiatric hospitalization; (2) PANSS score increase ≥25%, or PANSS score increase ≥10 if baseline PANSS score is below 40; (3) The concept or behavior of suicide.

### Statistical Analyses

The measurement data of normal distribution were expressed as mean ± standard deviation, and the comparison among groups was conducted by one-way analysis of variance. The measurement data of abnormal distribution were described as mean ± standard deviation or median and its quartile. The Mann-Wallis test was used for comparison among groups of independent samples, and the Mann-Whitney U test was used for pairwise comparison. Analysis of variance and analysis of covariance were used for comparison between groups.

Kaplan-Meier survival curves were used to draw the TDR curves at each time point. Cox proportional hazard regression model was used to assess the relative risk of TTD of antipsychotics. The log rank test was used for comparison between groups. If the overall difference was statistically significant, the Hochberg method was used to adjust the test level for multiple comparisons, and the *p* value was 0.0083 (0.05/6)(Huque, 2016). All statistical tests were performed with two-sided test, and *p* ≤ 0.05 was considered to be statistically significant. SPSS 22.0 software was used for analysis, and the graphical expression was completed by Graphpad Prism 8.

## Results

### Sample Description

**FIGURE 1:**
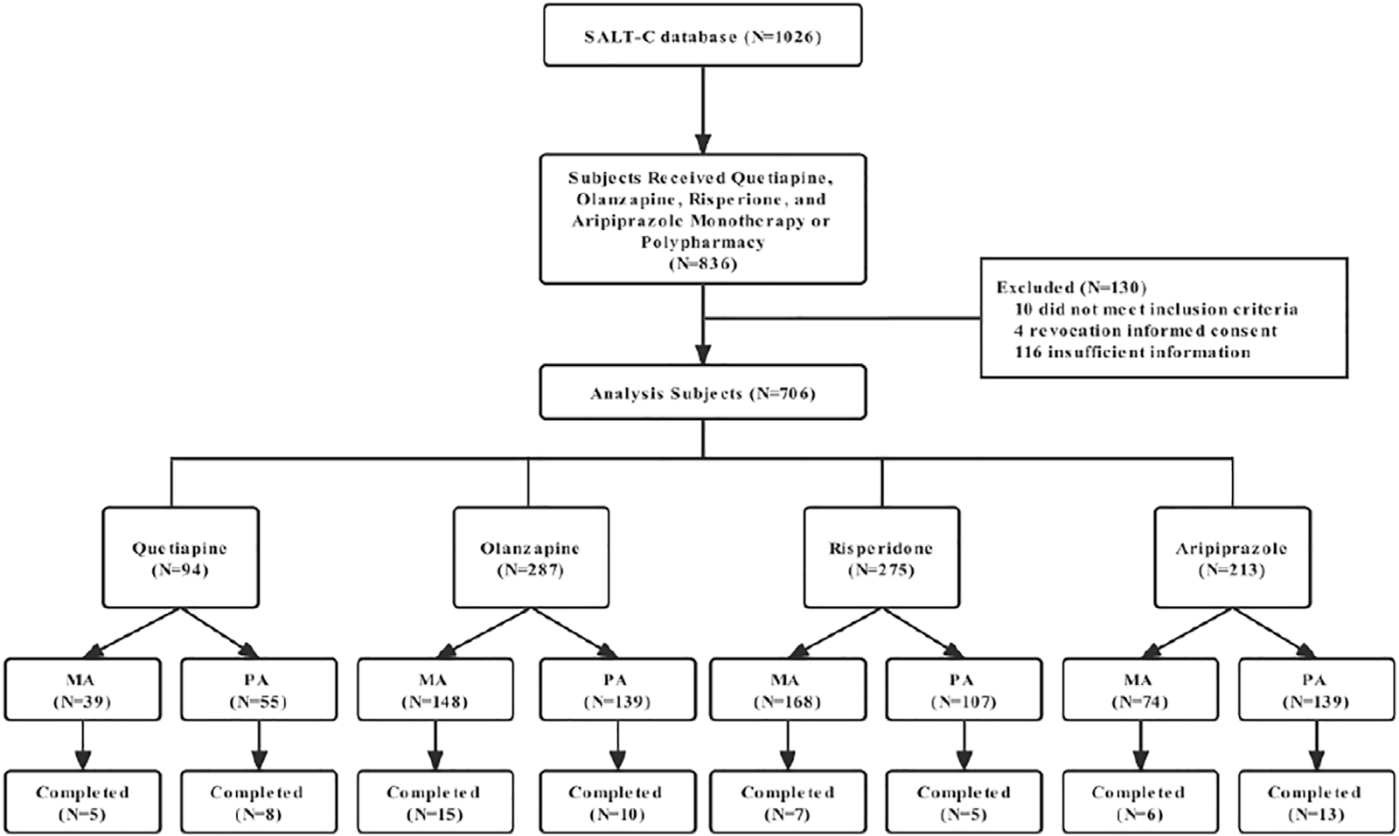
Consort diagram showing flow of subjects in the study.

From July 2011 to January 2017, a total of 1,026 patients with schizophrenia were collected. 836 received quetiapine, olanzapine, risperidone, and aripiprazole monotherapy or polypharmacy. Among them, 10 patients did not meet the inclusion criteria as typical antipsychotic drugs were used in combination, four patients withdrew their informed consent after enrollment, 116 patients were excluded due to a large number of missing data (Figure 1). In the cohort, including 706 patients, 405 individuals (57.3%) were men, and the mean age was 37.8 years. There was no significant difference in age between monotherapy group (*p* > 0.05). More female patients took aripiprazole, olanzapine, or quetiapine, whereas risperidone did the opposite in monotherapy group and there was no significant differences (*p* > 0.05). Aripiprazole was found to be used more male than female (*p* > 0.05). At 3 years of follow-up, monotherapy accounted for 26.8% of patients with first-episode schizophrenia, but only 1.9% with polypharmacy therapy. Demographic and clinical characteristics of patients are shown in Table 1; Table 2.

**TABLE 1:** Demographic and clinical characteristics of the study population (MA).

	Quetiapine MA (*n* = 39)	Olanzapine MA (*n* = 148)	Risperidone MA (*n* = 168)	Aripiprazole MA (*n* = 74)	*p*
Age/years	42.6 ± 16.2	36.4 ± 14.9	41.0 ± 13.5	32.1 ± 12.3	<0.001
Gender/male,n (%)	16 (41.0)	63 (43.8)	86 (53.8)	26 (37.7)	0.11
Race/Han,n (%)	31 (79.5)	123 (83.1)	163 (97.0)	63 (85.1)	<0.001
Age at onset/year	26.1 ± 11.0	26.9 ± 10.0	27.9 ± 9.7	25.7 ± 9.9	0.40
Total illness duration/years	16.6 ± 14.1	9.5 ± 12.3	13.1 ± 11.6	6.4 ± 7.4	<0.001
Duration of the illness/years	1.6 ± 2.4	1.7 ± 3.5	2.0 ± 3.8	1.2 ± 1.8	0.38
Dose/equivalent dose of olanzapine	11.0 ± 5.32	12.5 ± 6.8	6.6 ± 2.6	8.47 ± 3.67	<0.001
DUP/months	8.99 ± 20.72	47.56 ± 172.39	32.9 ± 95.77	73.57 ± 258.16	0.15
First episode,n (%)	6.0 (15.4)	52 (33.5)	37 (22.0)	19 (25.7)	0.02
Number of onset	4.5 ± 3.5	3.0 ± 2.7	3.4 ± 2.7	2.6 ± 2.0	0.002
PANSS	65.9 ± 20.3	69.5 ± 20.0	66.6 ± 29.5	64.8 ± 19.1	0.51
Total score	15.0 ± 7.8	16.8 ± 6.8	17.5 ± 8.2	14.9 ± 5.0	0.03
Positive score	17.9 ± 7.9	18.1 ± 6.4	19.7 ± 7.6	17.1 ± 7.0	0.04
Negative score	33.0 ± 10.5	34.5 ± 11.1	34.8 ± 11.3	32.8 ± 9.7	0.51
General psychopathology Score					
PSP	67.3 ± 20.5	50.8 ± 15.0	53.0 ± 15.2	58.5 ± 22.5	<0.001

DUP, Duration of Untreated Psychosis; MA, monotherapy of antipsychotic.

**TABLE 2:** Demographic and clinical characteristics of the study population (PA).

	Quetiapine PA (*n* = 55)	Olanzapine PA (*n* = 139)	Risperidone PA (*n* = 107)	Aripiprazole PA (*n* = 139)
Age/years	41.5 ± 15.6	33.6 ± 14.8	39.5 ± 14.4	33 ± 13.2
Gender/male,n (%)	23 (41.8)	62 (44.6)	49 (45.8)	80 (57.6)
Race/Han,n (%)	53 (96.4)	123 (73.2)	93 (86.9)	128 (92.1)
Age at onset/year	24.1 ± 12.0	23.0 ± 8.1	26.1 ± 12.0	26.1 ± 11.0
Total illness duration/years	17.6 ± 14.1	10.1 ± 13.1	16.6 ± 14.1	16.6 ± 14.1
Duration of the illness/years	2.6 ± 4.3	2.2 ± 4.8	3.2 ± 5.5	2.7 ± 5.3
Dose/equivalent dose of olanzapine	9 ± 5.04	12.6 ± 6.4	6.4 ± 3.2	9.73 ± 5.13
DUP/months	9.96 ± 20.72	36.67 ± 164.57	36.67 ± 31.24	103.34 ± 164.57
First episode,n (%)	4.0 (7.3)	37 (26.6)	14 (13.1)	30 (21.6)
Number of onset	5.0 ± 2.7	3.1 ± 3.1	3.7 ± 2.7	3.0 ± 2.1
PANSS	64.2 ± 19.8	65.2 ± 20.1	64.6 ± 20.6	55.1 ± 25.1
Total score	15.0 ± 6.8	14.5 ± 6.7	13.9 ± 6.7	13.1 ± 5.7
Positive score	17.7 ± 8.4	17.8 ± 8.4	119.8 ± 8.4	17.3 ± 8.2
Negative score	31.4 ± 9.2	30.8 ± 9.1	31.0 ± 10.3	30.4 ± 9.3
General psychopathology Score				
PSP	68.1 ± 19.5	55.0 ± 16.3	67.3 ± 20.5	60.6 ± 20.1

DUP, Duration of Untreated Psychosis; PA, polypharmacy of antipsychotic.

**FIGURE 2:**
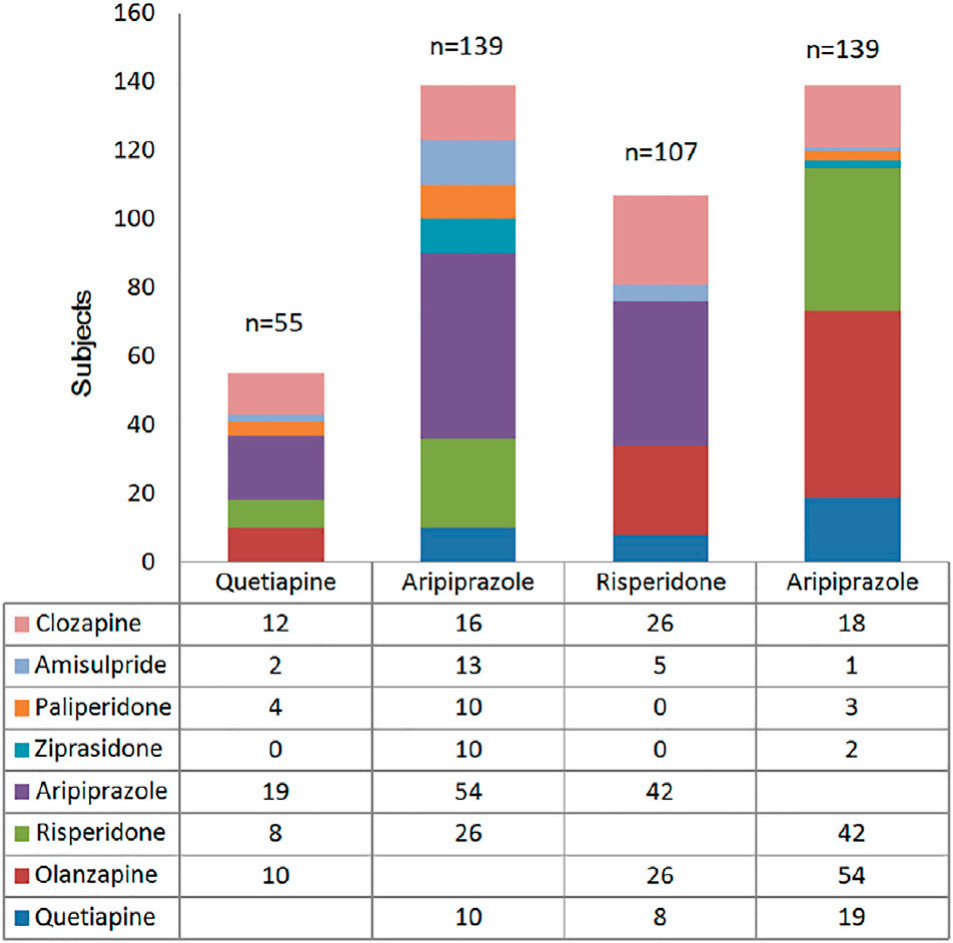
Analysis of Polypharmacy of antipsychotic.

Risperidone was the most commonly used in monotherapy, with 168 patients. The most commonly polypharmacy was the combination of olanzapine and aripiprazole, with 54 patients, followed by aripiprazole and risperidone with 42 patients. The results were shown in Figure 2.

SALT-C, An Observational Safety and Related Factors Study on Atypical Antipsychotics Long-term Treatment in Chinese Patients with Schizophrenia; MA, monotherapy of antipsychotic; PA, Polypharmacy of antipsychotic.

### Physical Condition and Concomitant Medications

A total of 351 patients (40.2%) were treated with medications other than atypical antipsychotics, among which quetiapine concomitant with medications accounted for the largest proportion [57 (60.6%)], aripiprazole concomitant with medications accounted for the smallest proportion [73 (34.3%)]. Risperidone concomitant with anticholinergic medications accounted for the largest proportion [60 (21.8%)], quetiapine concomitant with antihypertensive medications accounted for the largest proportion [13 (13.8%)]. The results were shown in Table 3.

**TABLE 3:** Concomitant medications of four antipsychotics.

Medication	Anticholinergic (%)	Anti insomnia (%)	Antidepressant (%)	Antihypertensive (%)	Hypoglycemic (%)	Total (%)
Quetiapine (*n* = 94)	9 (9.6)	5 (5.3)	6 (6.4)	13 (13.8)	6 (6.4)	57 (60.6)
Olanzapine (*n* = 287)	14 (4.9)	5 (1.7)	19 (6.6)	10 (3.5)	13 (4.5)	99 (34.5)
Risperidone (*n* = 275)	60 (21.8)	32 (11.6)	18 (6.5)	18 (6.5)	21 (7.6)	122 (44.4)
Aripiprazole (*n* = 213)	10 (4.7)	29 (13.6)	10 (4.7)	20 (9.4)	19 (8.9)	73 (34.3)

## TDR and TTD

### Comparison of Four Atypical Antipsychotics in TDR and TTD With Monotherapy

**FIGURE 3:**
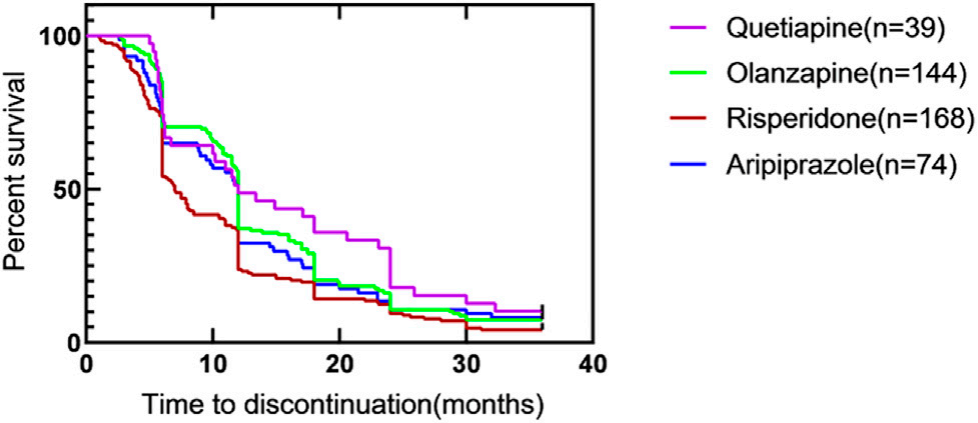
Time to all-cause discontinuation for four kinds of atypical antipsychotics with monotherapy, Kaplan–Meier survival curve^#.^ #. Survival curves are estimated on the basis of the observed raw data using the non-parametric Kaplan–Meier approach.

The corresponding results of Kaplan-Meier survival curves for TTD with monotherapies were shown in Figure 3. There was a significant difference among groups in all-cause antipsychotic treatment discontinuation (*p* = 0.0057). Among the four medications, the TDR of risperidone was the highest, and it was statistically significant. There were differences between quetiapine and risperidone (*p* = 0.033), olanzapine and risperidone (*p* = 0.003). The TDR at different time points were shown in Table 4.

**TABLE 4:** Treatment discontinuation at different time points.

Time	Quetiapine	Olanzapine	Risperidone	Aripiprazole
3 months/n (%)				
MA	0 (0)	5 (3.4)	12 (7.1)	5 (6.8)
PA	0 (0)	16 (11.5)	17 (14.5)	9 (6.5)
6 months/n (%)				
MA	8 (20.5)	44 (29.7)	77 (45.8)	26 (35.1)
PA	14 (25.5)	69 (49.6)	54 (46.1)	50 (36.0)
12 months/n (%)				
MA	19 (48.7)	93 (62.8)	128 (76.2)	50 (67.6)
PA	31 (56.4)	101 (72.7)	92 (78.6)	83 (59.7)
18 months/n (%)				
MA	23 (59.0)	118 (79.2)	144 (85.7)	60 (81.1)
PA	38 (69.1)	114 (82.0)	104 (88.9)	101 (72.7)
24 months/n (%)				
MA	27 (69.2)	132 (89.2)	152 (90.5)	66 (89.2)
PA	44 (80.0)	123 (88.5)	108 (92.3)	112 (80.6)
30 months/n (%)				
MA	33 (84.6)	136 (91.9)	160 (95.2)	67 (90.5)
PA	46 (83.6)	126 (90.6)	111 (94.9)	122 (87.8)
36 months/n (%)				
MA	35 (89.7)	137 (92.6)	161 (95.8)	68 (91.9)
PA	47 (85.5)	132 (95.0)	113 (96.6)	126 (92.0)

MA, monotherapy of antipsychotic; PA, polypharmacy of antipsychotic.

### Cox Regression Analysis

The cox regression analysis of TTD in four groups of monotherapy were shown in Table 5. The relative risk of quetiapine was less than risperidone (*p* = 0.012).The relative risk of olanzapine was also lower than risperidone (*p* = 0.006). However, after correction of Hochberg with multiple tests, only olanzapine had a relative risk lower than risperidone (*p* < 0.0083).

**TABLE 5:** Cox regression analysis of TTD in four groups of monotherapy.

Group (n)		Olanzapine (*n* = 148)	Risperidone (*n* = 168)	Aripiprazole (*n* = 74)
Quetiapine (*n* = 39)	HR	1.158	1.596	1.263
	95% CI	0.798–1.680	1.106–2.303	0.839–1.900
	*P*	0.441	**0.012** ^#^	0.263
Olanzapine (*n* = 148)	HR		1.379	1.090
	95% CI		1.097–1.732	0.815–1.458
	*P*		**0.006** ^*^	0.561
Risperidone (*n* = 168)	HR			0.794
	95% CI			0.598–1.055
	*p*			0.112

* The relative risk of olanzapine was also lower than risperidone (p = 0.006). Statistically significant differences after Hochberg correction (*p* < 0.0083). #: The relative risk of quetiapine was less than risperidone (*p* = 0.012).

### Comparison of TDR and TTD Between Monotherapy and Polypharmacy

**FIGURE 4:**
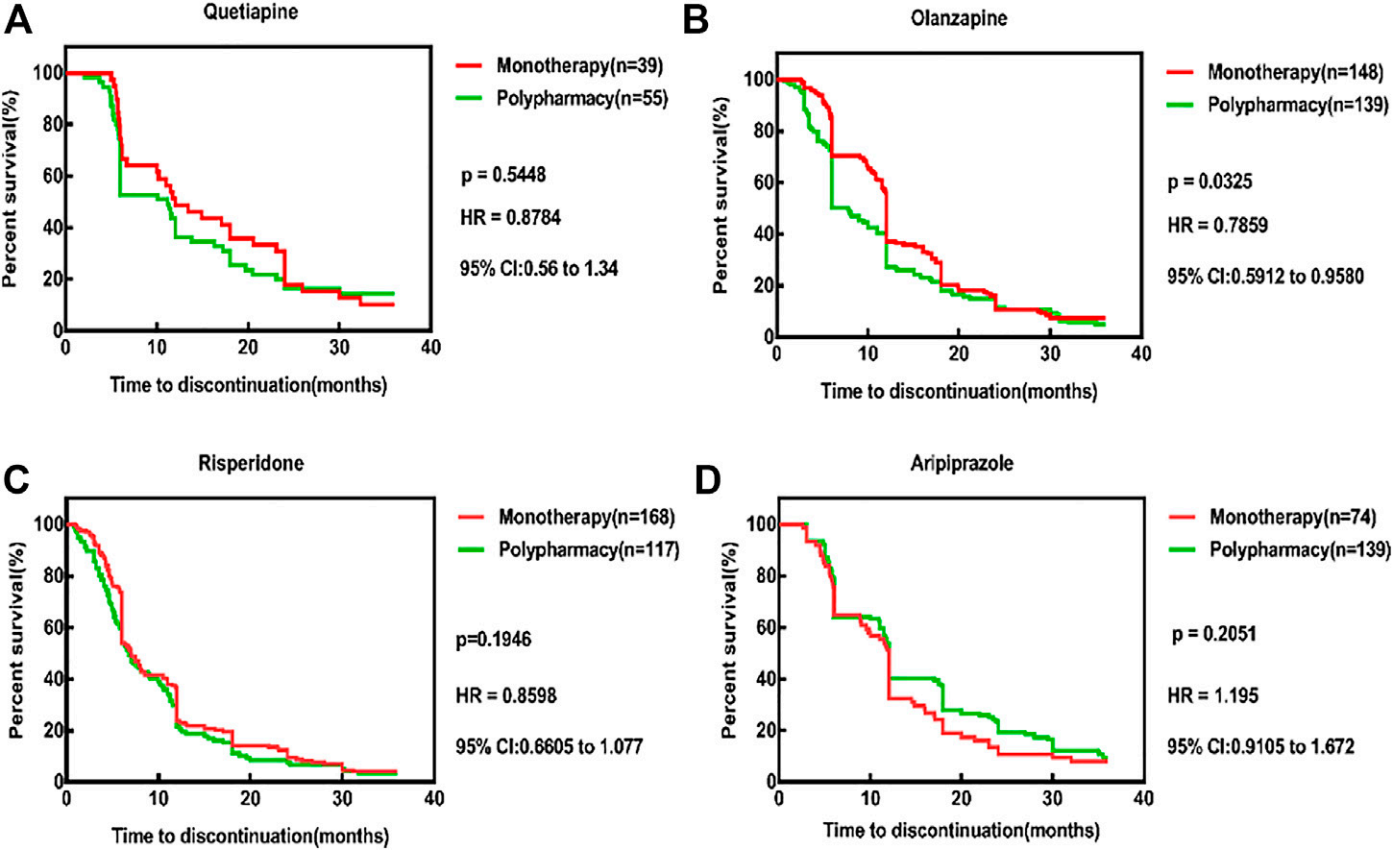
Time to all cause discontinuation for monotherapy and polypharmacy, Kaplan-Meier survival curves. **(A)** The TDR of quetiapine monotherapy was lower than that of polypharmacy (*p* = 0.5448, HR = 0.8784, 95% CI: 0.56–1.34). **(B)** The TDR of olanzapine monotherapy was lower than that of polypharmacy and statistically different (*p* = 0.0325, HR = 0.7859, 95% CI: 0.5912–0.9580). **(C)** The TDR of risperidone monotherapy was lower than that of polypharmacy (*p* = 0.1916, HR = 0.8598, 95% CI: 0.6605–1.077). **(D)** The TDR of ariprazole monotherapy was higher than that of polypharmacy (*p* = 0.2051,HR = 1.195, 95% CI: 0.9105–1.672). Survival curves are estimated on the basis of the observed raw data using the nonparametric Kaplan–Meier approach.

The comparison of TTD between monotherapy and polypharmacy were shown in Table 6. The overwhelming majority of comparisons demonstrate longer time to discontinuation for monotherapy than for polypharmacy, as indicated by the survival distributions. The results showed that compared with the other three medications, risperidone had the shortest TTD in both monotherapy group and polypharmacy group (7.0 and 6.8 months). At 12, 24, and 36 months, the TDR of risperidone was higher than that of other groups. After 24 months of aripiprazole, the TDR of the polypharmacy was lower than that of the monotherapy. Compared with polypharmacy, except for aripiprazole, the TDR of other three monotherapy medications were lower than that of polypharmacy, and olanzapine was statistically different (*p* = 0.0325). The results were shown in Figure 4.

**TABLE 6:** Comparison of TTD among four atypical antipsychotics.

	TTD/months	Mean	95%CI	Median	95%CI
Quetiapine	MA	16.0	12.8–19.3	12.0	7.2–16.8
	PA	14.1	11.1–17.1	11.2	6.0–12.0
Olanzapine	MA	14.1	12.7–15.5	12.0	11.9–12.0
	PA	11.5	9.9–13.1	7.8	6.0–11.0
Risperidone	MA	10.9	9.6–12.2	7.0	6.5–7.5
	PA	9.8	8.3–11.3	6.8	6.0–9.0
Aripiprazole	MA	13.2	11.1–15.4	12.0	11.5–12.5
	PA	15.3	13.5–17.1	12.0	11.5–12.0

MA, monotherapy of antipsychotic; PA, Polypharmacy of antipsychotic; CI, confidence interval.

## Discussion

At present, there is a great controversy about the efficacy of different antipsychotics in the treatment of schizophrenia. The choice of antipsychotic medications was mainly based on the overall effectiveness of medications, and there was still a lack of long-term controlled trials of multiple medications. In addition, most of these studies were randomized controlled trials (RCT) that screened patients with strict inclusion and exclusion criteria and could not represent the majority of patients in the real world. Moreover, the follow-up period of these trials was short, and the results of these trials cannot be inferred in the long-term treatment process. Therefore, observational studies are the only way to investigate long-term comparative outcomes (Tiihonen et al., 2017). This study was a real-world study of the long-term outcomes of atypical antipsychotics in the treatment of patients with schizophrenia, which reduced the limitations of traditional studies, reflected the clinical efficacy of therapeutic drugs in the real world, truly reflected the specific details of clinical practice, and the conclusions of the study can be directly applied to the clinical practice.

The results of this study showed that the average TDR of monotherapy for 18 months was 80.4%, the average TDR of polypharmacy was 81.1%, the average TDR of monotherapy for 3 years was 93.5%, and the average TDR of polypharmacy was 95.0%. In the CATIE study, the TDR within 18 months was 75%, and the median TTD was about 6 months (Lieberman et al., 2005). The reason why the TDR of our study was higher than that was due to the difference in study design. We adopted a more conservative method in defining all-cause treatment discontinuation as we included patients with schizophrenia lost to follow-up. The relapse rate was 18% in those who discontinued antipsychotics, which was much lower than other study (Üçok and Kara, 2020). The reson was as a real-world study, the result was based on patients who completed follow-up, whereas for those who were lost to follow-up, we lost this information, so this data may be skewed. A 3-years follow-up randomized clinical trial of patients with first-episode psychosis showed that the TDR reached 81.7% (Gómez-Revuelta et al., 2018), which also illustrated the adherence of patients with schizophrenia was not as good as other chronic diseases (Tiihonen et al., 2018). In addition, the results of this study showed that after correction of Hochberg with multiple tests, olanzapine had a lower risk than risperidone, which means the TDR of olanzapine was superior to the other three antipsychotics, which was consistent with previous studies. A meta-analzysis compared the effectiveness of olanzapine and other antipsychotics in the treatment of schizophrenia and the results showed that on time to all-cause medication discontinuation, olanzapine was significantly better than all other atypical antipsychotics except clozapine (Soares-Weiser et al., 2013; Zhang et al., 2013). A meta-analysis showed that regarding all-cause discontinuation, olanzapine was significantly superior to risperidone, quetiapine and aripiprazole (Kishimoto et al., 2019). Besides, the results of this study showed that the TDR of risperidone was higher than other medications. A review included 45 blinded RCTs showed that risperidone improved the general mental state slightly less than olanzapine and risperidone was also less efficacious than olanzapine and clozapine. Furthermore, risperidone produced more extrapyramidal side effects than other antipsychotics and increased prolactin levels clearly more than all comparators (Komossa et al., 2011). Although we did not analyze the reasons for the TDR, a large proportion of risperidone combined with anticholinergic drugs prompting adverse reactions may be one of the reasons for the high discontinuation rate of risperidone. Quetiapine was the least successful monotherapy, as has been observed also in previous Swedish and Finnish studies (Tiihonen et al., 2017; Taipale et al., 2018).

There were 283 cases of polypharmacy with two or more atypical antipsychotics, accounting for 40.08%, showing the prevalence of antipsychotic polypharmacy had been increasing in real-world clinical settings. Several database studies utilising health claims data in the United States reported antipsychotic polypharmacy prevalence rates from 4.6 to 23% (Kreyenbuhl et al., 2006; Morrato et al., 2007). Antipsychotic polypharmacy prevalence rates reported from Asia were often higher, ranging from 17.8 to 51.7% (Hou et al., 2016; Kochi et al., 2017). In addition, studies have shown that antipsychotic polypharmacy was used clinically in patients with severe schizophrenia, especially those with mania or with violent or aggressive behaviours, or to avoid side-effects resulting from high doses of monotherapy (Jeon and Kim, 2017). Antipsychotic polypharmacy may also be used in treatment-resistant schizophrenia, such as clozapine in combination with atypical antipsychotics (Galling et al., 2017; Tiihonen et al., 2019). However, there was still a lack of strong evidence to support the effectiveness of these programs. Besides, this study concluded that olanzapine monotherapy was superior to polypharmacy, which was consistent with the results of some other studies (Novick et al., 2012; Katona et al., 2014). A nationwide study in Hungary showed that the median times to all-cause discontinuation of olanzapine for monotherapy and polypharmacy were 222 and 86 days, respectively, showing monotherapy was superior to polypharmacy for long-term sustained treatment (Katona et al., 2014). The Canadian Psychiatric Association expressly stated that there is no evidence to support the polypharmacy of 2 or more antipsychotics to improve efficacy. The World Federation of Biological Psychiatry also proposed to use monotherapy as much as possible. The National Institute of Health and Nursing in the United Kingdom also advocated the principle of monotherapy for the treatment of schizophrenia. The Chinese guidelines for the prevention and treatment of schizophrenia also proposed that drug treatment should be initiated. According to the manifestation of clinical symptoms, an atypical antipsychotic drug can be selected for treatment. The principle of monotherapy should be used. If the therapeutic dose was not effective, the dosage should be increased or consider another medication, still treated with monotherapy. Although some studies have found that the polypharmacy of some antipsychotics had better efficacy, and the combination of antipsychotics and antidepressants had better efficacy in the treatment of patients with schizophrenia (Helfer et al., 2016). Studies have also found that benzodiazepines can enhance the efficacy of antipsychotics (Włodarczyk et al., 2017), and that combined with lithium can enhance the efficacy and improve negative symptoms (Muñoz-Negro et al., 2019). However, the polypharmacy of antipsychotics has not been recognized, and first-line treatment is not recommended. In view of the low quality of the evidence and the lack of double-blind and high-quality evidence of effectiveness, these conclusions should be considered preliminary and inconclusive. This study did not find that the polypharmacy of antipsychotics can significantly improve the TDR and TTD of patients. Therefore, a larger sample size is needed for future studies to master the knowledges of adverse drug reactions and drug interactions in polypharmacy, which is conducive to the rational clinical use of antipsychotics.

This study was an open observational study conducted the long-term efficacy of antipsychotic medications in real-world settings. The inclusion and exclusion criteria were wide, and certain medications were allowed to be combined. Therefore, the results of this study can better reflect the clinical practice and its application will be more extensive. However, several limitations should be considered when interpreting our results. First, although we used scales to assess patients’ symptoms, such as PANSS to classify recurrence (Supplementary Figure S2), data were still missing for patients who were lost to follow-up. Therefore, the study did not obtain detailed data on whether discontinuation of antipsychotics due to resistance or ineffectiveness. In addition, more than two atypical antipsychotics simultaneously or combination with typical antipsychotic drugs were not investigated in the analysis due to their low prevalence, which restricts generalizability. Third, patients may be subjected to monotherapy or polypharmacy strategies based on clinical or demographic characteristics and prior disease history, so the monotherapy and polypharmacy of antipsychotic groups might show baseline differences. We conducted propensity score adjusted approaches to alleviate selection bias and the results remain essentially unchanged after matching the baseline characteristics (see Supplementary Table S1). Nevertheless, propensity score adjusted method in a naturalistic study can control only for a limited number of covariates and cannot account for the unmeasured ones.

## Conclusion

Risperidone monotherapy had the highest TDR and the shortest TTD. This study also observed a large proportion of risperidone combined with anticholinergic drugs. This suggested that risperidone may be associated with more extrapyramidal adverse reactions. Olanzapine monotherapy had a relative risk lower than risperidone and was superior to polypharmacy.

## Data Availability

The raw data supporting the conclusions of this article will be made available by the authors, without undue reservation.
