# Supplementary material for: Long-Term Antipsychotic Effectiveness and Comparison of the Efficacy of Monotherapy and Polypharmacy in Schizophrenia: A 3-Years Follow-Up “Real World” Study in China
Source: Front Pharmacol. 2022 Jun 13;13:860713. doi: 10.3389/fphar.2022.860713 (PMC9234304; doi:10.3389/fphar.2022.860713)
Supplement: Supplementary file 1 [file DataSheet1.docx]

Supplementary Material

| B  A |  |
| --- | --- |

Figure S1 The analysis of all the centers and drug use

A）Enrollment of each center, 1 Shanghai Mental Health Center; 2 Sixth Hospital of Peking University; 3 Beijing Anding Hospital; 4 Guangzhou Psychiatric Hospital; 5 West China Hospital of Sichuan University; 6 Second Xiangya Hospital; 7 First Affiliated Hospital of Kunming Medical College，8 Shanghai Luwan Mental Health Center；

B）Drug use，MA：monotherapy of antipsychotic；PA：Polypharmacy of antipsychotic.


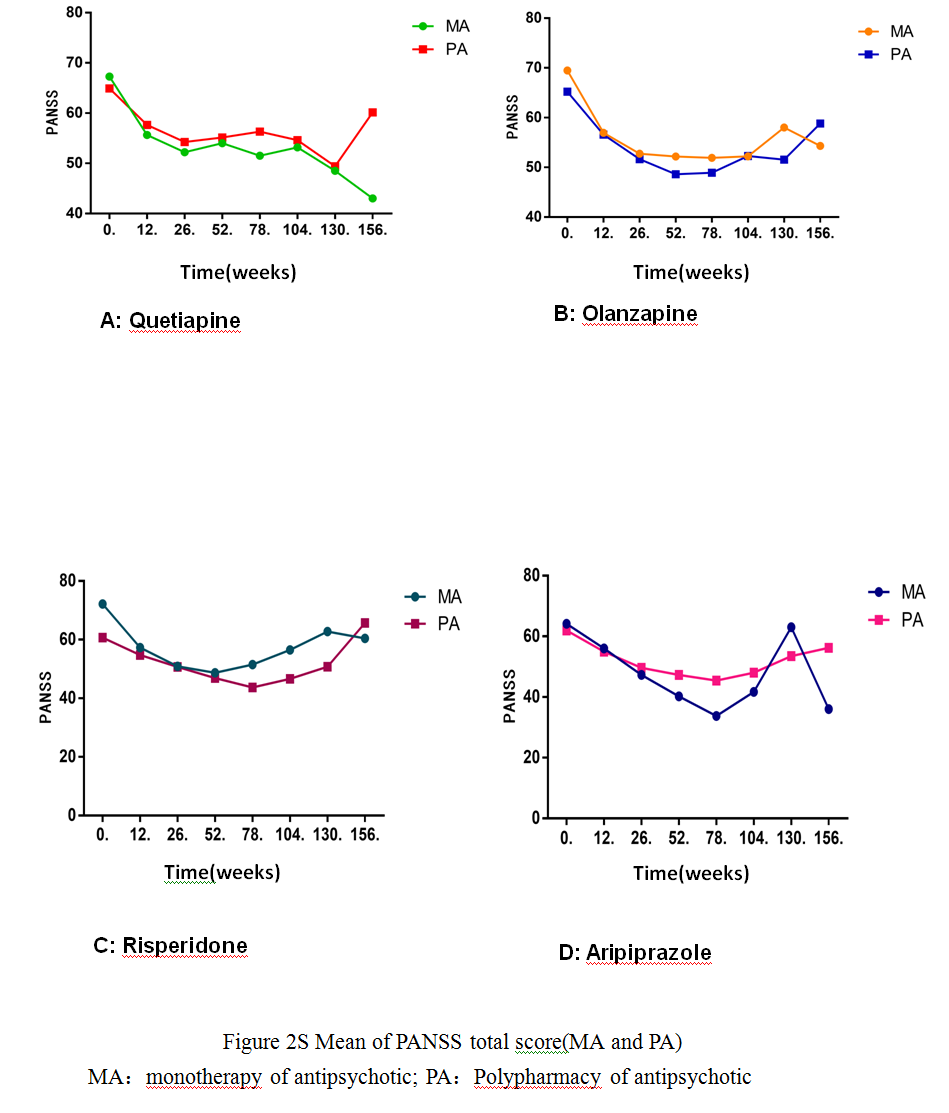


Table S1 Hazard ratio estimates for all-cause discontinuation

|  | Raw |  | Adjusted# |  |
| --- | --- | --- | --- | --- |
| Medications | HR | *p* | HR | *p* |
| Quetiapine | 0.88 | 0.5448 | 0.82 | 0.5012 |
| Olanzapine | 0.79 | 0.0325* | 0.78 | 0.0169* |
| Risperidone | 0.86 | 0.1946 | 0.86 | 0.2923 |
| Aripiprazole | 1.20 | 0.2051 | 1.16 | 0.4537 |

Hazard ratio and *p* value were adjusted based on propensity scores for matched-pair data. Specific variables used for the matching : Quetiapine：Race. Olanzapine : General psychopathology Score. Risperidone：Age at onset；Number of onset；Duration of the illness；Positive score. Aripiprazole：Age；Total illness duration；Duration of the illness；Negative score；PSP.
